# “Niguas,” a Parasite of Northern Mexico

**Published:** 1886-12

**Authors:** C. K. Gregg

**Affiliations:** Ramos Arispe, Mexico


### "NIGUAS," A PARASITE COMMON TO NORTHERN MEXICO.

*By C. K. Gregg, M. D., Ramos Arispe, Mexico.*

(For Daniel's Medical Journal.)

MY attention was first drawn to the troublesome and loathsome condition produced by "niguas" by a case seen with my friend, Dr. R. H. L. Bibb, in Saltillo two years ago. Neither of us at that time had ever seen or heard of such an affection, and we were forced to regard the case as one of syphilitic ulcers of the foot, notwithstanding the fact that there existed no well marked syphilitic history, the patient being a young woman of the poorest class of natives. However, we lost sight of the case after having put the patient on syphilitic treatment. Several months elapsed before I saw another case, and upon close inquiry I found the disease to be very common, especially—I may say almost exclusively—among the lower class of Mexicans. This condition of things, which gives rise to so much suffering, and which is so rebellious to treatment, is due to the entrance under the skin of "niguas," as they are called by the natives, or which are better known to Americans, and those especially of the Southern States, as "jiggers," or flesh worms, a most uninviting little insect, which produces such ravages when once it gets upon, and makes its way under, the skin of a human being. Their seat of prediliction is the feet, more especially under the toe nails and inter-digital spaces; their entry almost exclusively at this part of the body is due to the fact that the poorer class of people, or "pelados," seldom apply soap and water to their pedal extremities, and never wear socks or stockings, and the majority only sandals instead of shoes. One can readily imagine, therefore, how easy it is for the insects to gain admittance to a part so greatly exposed and neglected as the feet. Hogs and other animals, I find upon inquiry, are known also to have large numbers of "niguas" between their toes. The insect presents the appearance of the nit, or the egg of the louse, only being somewhat larger. I regret that I cannot give the microscopical appearance. The insect first manifests itself by an itching or uneasy sensation at or near the toes or under the nails, which, by scratching, becomes inflamed and gives rise to ulcers, with more or less destruction of flesh, in some cases the bones being almost denuded. If treatment is commenced early, before much loss of tissue has taken place, the evil can generally be easily remedied, but when considerable time has elapsed, the disease tends to become chronic in character and difficult to relieve; in some cases the whole foot is swollen and even abcesses occur, as I have seen in a few instances, on the palm or surface. The patient in such a condition as this is of course strictly confined to the bed, and needs constitutional, as well as local, treatment. As a rule careful cleansing and mild local applications is alone necessary, Iodoform or oxid. zinc, in the form of the dry powder, being more efficacious than ointments. From two to three weeks is the time required in mild cases before granulation can take place, on the contrary, severe cases may require several months.

I have taken the liberty of trespassing upon the space of your valuable journal, as, prior to practicing in Mexico, I had never heard of such an affection, and deem it of sufficient interest to call the attention of your readers to it, that I may be further enlightened on the subject.

